# Efficacy of Oral Mucosal Grafting for Nasal, Septal, and Sinonasal Reconstruction: A Systematic Review of the Literature

**DOI:** 10.3390/life15081281

**Published:** 2025-08-13

**Authors:** Marta Santiago Horcajada, Alvaro Sánchez Barrueco, William Aragonés Sanzen-Baker, Gonzalo Díaz Tapia, Ramón Moreno Luna, Felipe Villacampa Aubá, Carlos Cenjor Español, José Miguel Villacampa Aubá

**Affiliations:** 1 Rhinology Unit, ENT and Cervicofacial Surgery Department, Fundación Jiménez Díaz University Hospital, 28040 Madrid, Spain; marta.shorcajada@quironsalud.es (M.S.H.); william.aragones@quironsalud.es (W.A.S.-B.); gdiazt@quironsalud.es (G.D.T.); carlos.cenjor@gmail.com (C.C.E.); jmvillacampa@fjd.es (J.M.V.A.); 2 Rhinology Unit, ENT and Cervicofacial Surgery Department, Villalba General University Hospital, 28400 Madrid, Spain; 3 Medicine Faculty, Universidad Alfonso X el Sabio, 28691 Villanueva de la Cañada, Spain; 4 Rhinology Unit, Department of Otolaryngology, Head and Neck Surgery, Virgen Macarena University Hospital, 41009 Seville, Spain; ramoluorl@gmail.com; 5 Continuing Education Master’s Program in Advanced Rhinology and Anterior Skull Base, International University of Andalucía, 41092 Seville, Spain; 6 Department of Urology, Clínica Universidad de Navarra, 28027 Madrid, Spain; fvillacampa@yahoo.es; 7 Medicine Faculty, Universidad Autónoma de Madrid, 28029 Madrid, Spain

**Keywords:** oral mucosa, autologous graft, nasal reconstruction, septal reconstruction, sinonasal reconstruction, skull base reconstruction, systematic review

## Abstract

**Background**: Reconstruction of nasal, septal, and nasosinusal defects is challenging when the native mucosa is absent or damaged. Oral mucosal grafts have been proposed as a reconstructive option due to their favorable biological properties, but their use in rhinology remains poorly defined. **Objective**: To evaluate the clinical efficacy and technical characteristics of oral mucosal grafting for nasal, septal, nasosinusal, and skull base reconstruction. **Data Sources**: PubMed, Embase, Web of Science, and Cochrane Library were searched for studies published between January 2005 and May 2025. **Study Eligibility Criteria**: We included original human studies (case reports or series) reporting the use of free or pedicled oral mucosal grafts in nasal, septal, nasosinusal, or skull base reconstruction. Non-original studies, animal or preclinical studies, and articles not in English or Spanish were excluded. **Methods of Review**: One reviewer screened titles, abstracts, and full texts using Rayyan. Methodological quality was assessed using JBI tools for case reports and case series. A narrative synthesis was conducted due to clinical heterogeneity and absence of comparison groups. The resulting assessments were reviewed by the co-authors to confirm accuracy and resolve any potential discrepancies. **Results**: Of 467 records identified, 10 studies were included. All were case reports or series involving buccal, palatal, or labial mucosa. Most reported good graft integration, low complication rates, and favorable functional outcomes. No randomized studies or comparative analyses were found. **Limitations**: Included studies had small sample sizes, lacked control groups, and showed heterogeneous methods and follow-up. The certainty of evidence could not be formally assessed. **Conclusions**: Oral mucosal grafting is a promising reconstructive option in selected nasosinusal and skull base defects. However, stronger comparative studies are needed to determine its clinical superiority. **Registration**: This review was not registered in any public database.

## 1. Introduction

Defects involving the nasal cavity, septum, paranasal sinuses, or skull base pose a significant reconstructive challenge due to their location in a complex anatomical region with essential functions, including respiration, humidification of inspired air, olfaction, and facial aesthetics. These are usually caused by aggressive tumor resections [1,2,3], trauma [4,5], or complex surgical sequelae such as septal perforations [1,4,6] and extended mucosal resection secondary to systemic inflammatory diseases such as chronic rhinosinusitis with polyps [7]. More rare causes are congenital causes or burns [8]. In this context, the use of oral mucosal flaps and grafts offers a promising option for restoring the functional and structural integrity of the nasal cavity and paranasal sinuses and must aim to provide a stable, functional, and biocompatible solution.

The most common reconstructive options include sinonasal locoregional flaps, microvascular free flaps, autologous grafts, and, in some cases, synthetic biomaterials. Local flaps offer vascular support and good integration but require sufficient reconstructive surface and may be associated with donor site morbidity. Free grafts, on the other hand, can cover large defects, although in some cases they may be heterogeneous and less uniform [9]. Autologous mucosal grafts and flaps, such as those derived from oral mucosa, allow internal surfaces to be lined with biologically compatible tissue [10]. These have proven viable across multiple surgical specialties due to the similarity of their histological characteristics [11], which promote rapid healing through variable vascularization and allow for the harvesting of large surfaces with minimal donor site morbidity [11].

In otolaryngology and head and neck surgery, the use of oral mucosa as a free graft is an effective and versatile technique for nasal and extranasal reconstruction, particularly useful when the nasal mucosa is absent or damaged. Its application ranges from the repair of septal perforations [1,4] and complex nasal mucosal defects to gingival, maxillary, and facial reconstructions following tumor resection [1,2,3,4,12,13,14,15]. Additionally, this technique is notable for preserving nasal functionality, preventing cicatricial retraction, and maintaining airway patency, with favorable outcomes even in challenging areas such as the anterior skull base [16] and the lower airways [17].

Despite these prerogatives, the use of oral mucosal grafts in nasal, septal, sinonasal, and skull base reconstruction remains poorly documented, limited almost exclusively to case reports and small clinical series, in the absence of in-depth reviews. This restricts the development of clear recommendations regarding their indication and clinical utility. Therefore, the objective of this systematic review is to analyze the feasibility, efficacy, and safety of oral mucosal grafts as a reconstructive alternative for defects located in the nasal cavity, septum, paranasal sinuses, and skull base.

## 2. Materials and Methods

### 2.1. Systematic Review Design and Methodological Quality Assessment

A systematic review of the literature was conducted with a descriptive and exploratory approach, aimed at addressing a structured question based on the PICO model (population, intervention, comparator, outcomes). The review was designed in accordance with the PRISMA (Preferred Reporting Items for Systematic Reviews and Meta-Analyses) guidelines, with the objective of identifying and synthesizing the available evidence on the use of oral mucosal grafts and flaps in the reconstruction of nasal, septal, sinonasal, or skull base defects.

Given the clinical and specialized nature of the intervention under evaluation, observational studies, including individual case reports, were also included. A meta-analysis was not performed due to heterogeneity in study designs, surgical techniques, sample sizes, and reported outcomes, as well as the absence of comparator groups.

To assess the methodological quality of the included studies (n = 10), critical appraisal tools were applied according to the design of each publication. All studies, both individual case reports and case series, were evaluated using the JBI (Joanna Briggs Institute) Critical Appraisal Checklist for Case Reports and the JBI Critical Appraisal Checklist for Case Series, respectively. These tools are widely recognized and were selected for their specificity in evaluating descriptive study designs. The assessments were performed manually by the first author. No automation tools were used. The resulting assessments were reviewed by the co-authors to confirm accuracy and resolve any potential discrepancies.

### 2.2. PICO Question

The review was structured around a predefined PICO question that guided the entire methodological process. The population (P) included human patients undergoing nasal, septal, sinonasal, or skull base reconstruction. The intervention (I) consisted of the use of oral mucosal grafts or flaps (buccal, labial, or palatal). A comparison (C) was not applicable in this review, as the included studies lacked comparator groups; however, it would involve comparing oral mucosal grafts or flaps to other reconstructive techniques—whether autologous, heterologous, or synthetic—if such studies were available. The outcomes (O) of interest were clinical viability of the graft, associated complications, and assessment of functional and aesthetic efficacy in the treated defects.

### 2.3. Search Criteria and Databases

To ensure a comprehensive and systematic literature search, specific filters were applied from the onset in the selected databases, limiting results to studies published between January 2005 and May 2025. This time restriction was directly applied in PubMed, Web of Science, Embase, and the Cochrane Library. Full-text access was obtained through personal and institutional subscriptions, open-access links provided by publishers, or by using legal tools to locate openly accessible versions.

In PubMed, a free-text search strategy was used without MeSH operators, capturing broad and recent studies. The search formula applied was: oral mucosal graft AND (nasal OR septal OR sinus OR skull base OR sinonasal OR craniobasal). No filters were initially applied by study type to maximize the sensitivity of the search.

In Web of Science, a similar free-text search strategy was used with the following terms: TS = (oral mucosa graft) AND TS = (nasal OR septal OR sinus OR skull base OR sinonasal OR craniobasal). Results were filtered by year of publication (2005–2025).

In Embase, a combined strategy using both controlled vocabulary (Emtree) and free-text terms was applied to increase search sensitivity. The search formula used was oral AND (‘mucosa’/exp OR mucosa) AND (‘graft’/exp OR graft) AND ((nasal OR septal OR ‘sinus’/exp OR sinus OR ‘skull’/exp OR skull) AND (‘base’/exp OR base) OR sinonasal OR craniobasal) AND [2005–2025]/py.

In the Cochrane Library, the following search strategy was used: oral mucosa graft AND (nasal OR septal OR sinus OR skull base OR sinonasal OR craniobasal). All available document types were included, such as systematic reviews, protocols, and clinical trials.

The applied search strategy yielded 467 records across the four databases, ensuring comprehensive coverage of studies addressing our research question.

### 2.4. Inclusion and Exclusion Criteria

Studies were selected according to predefined inclusion and exclusion criteria established prior to the search. Eligible articles were original studies describing the use of oral mucosal grafts or flaps in nasal, septal, sinonasal, or skull base reconstruction procedures in human patients, regardless of age.

Case reports and clinical case series were included, with the condition that case series with fewer than five patients were accepted only if they focused directly on the objective of this review. This decision was made because, given the scarcity of the literature and limited available evidence on this technique, it was considered important to gather all relevant clinical experience to generate working hypotheses, despite the methodological limitations. Conversely, case series with fewer than five patients that did not specifically address the use of oral mucosa in the areas of interest were excluded during the initial screening phase, provided this information was clearly stated in the title or abstract.

In addition, non-original studies (such as reviews and letters to the editor), preclinical experimental studies, articles published in languages other than Spanish or English, and those focused on other reconstructive techniques unrelated to oral mucosa were excluded.

### 2.5. Data Extraction

Data extraction was performed manually by the first author. The following information was collected from each study: study design, year of publication, number of patients, patient demographics, anatomical site of reconstruction, type of oral mucosa used, graft or flap configuration, surgical technique, and follow-up duration.

The primary outcomes extracted were graft viability at the recipient site, complication rates, and donor site morbidity. Secondary outcomes included functional results and postoperative follow-up details. All available data on these outcomes were recorded, regardless of time points or assessment methods.

When relevant specific information was missing or unclear, it was recorded as “not reported”, and no assumptions were made. No automation tools were used for data collection. The final dataset was reviewed by the co-authors to ensure accuracy and completeness.

### 2.6. Screening

The study selection process was conducted in two consecutive phases, following the criteria defined in the review protocol. In Phase 1, an initial screening of titles and abstracts was performed to exclude records that were clearly irrelevant to the research question, specifically those with no or only tangential relation to the topic of interest. For example, studies addressing nasal surgery or oral mucosal grafts were excluded if they did not explicitly evaluate their use in sinonasal reconstruction. This step allowed the review to focus exclusively on studies that directly addressed the research objective, thereby ensuring greater relevance and quality in the subsequent analysis. Studies whose titles or abstracts suggested potential relevance were retained for full-text assessment in the next phase.

In Phase 2, the full texts of the preselected articles were reviewed in detail, with rigorous application of the previously defined inclusion and exclusion criteria (study type, population, intervention, language, and full-text availability).

Both screening phases were conducted independently and carried out by a single reviewer (the first author) using the Rayyan platform, which enabled the efficient organization of results and duplicate detection and facilitated decision making throughout the review process. The full manuscript and final inclusion list were later reviewed and validated by the co-authors to ensure consistency and methodological accuracy. No automation tools were used in the selection process.

**Figure 1:**
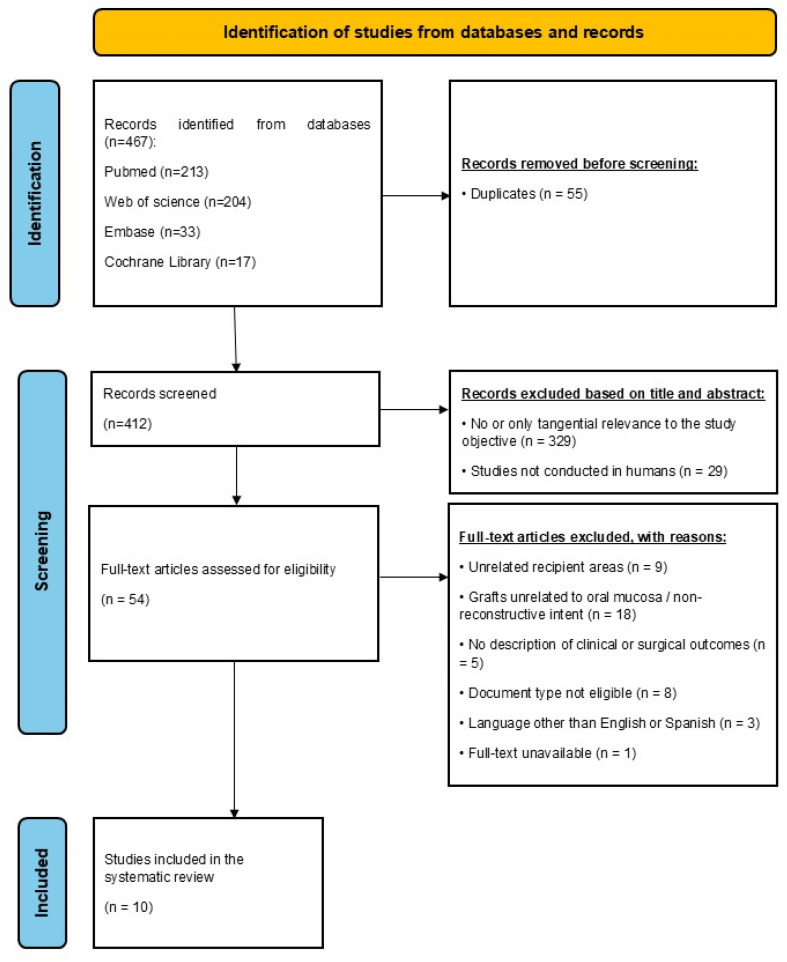
PRISMA flow diagram illustrating the study selection process for this systematic review.

The entire process of identification, screening, eligibility assessment, and final study selection is illustrated in the PRISMA flow diagram (Figure 1).

### 2.7. Data Synthesis

**Figure 2:**
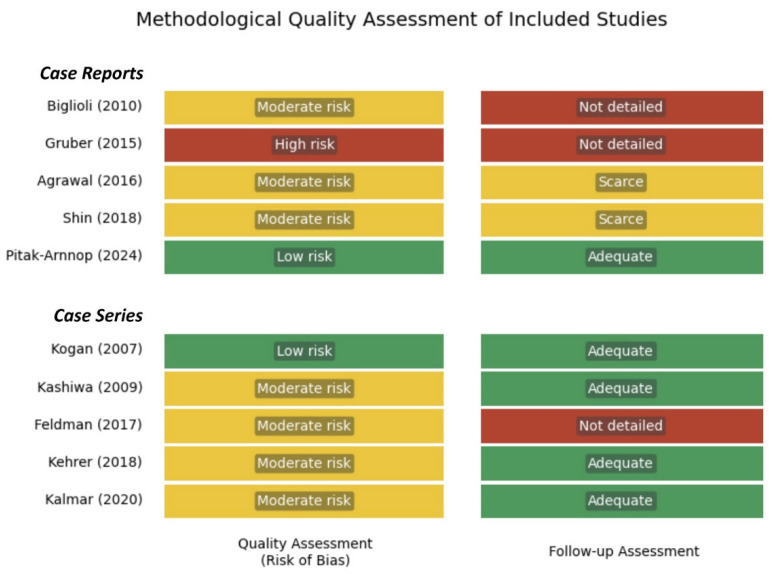
Methodological quality assessment of included studies in systematic review (using the JBI Case Series and Case Report Checklist).

No formal effect measures (e.g., relative risk, mean difference) were calculated due to the descriptive nature of the included studies and the absence of comparator groups. All data were synthesized narratively. No data transformation or conversion procedures were applied. All outcomes were reported as described in the original publications. Due to the heterogeneity of study designs (case reports or series) and outcomes, no data pooling or standardization procedures were performed prior to synthesis. Results from individual studies were organized in summary tables and figures to facilitate comparison. Study characteristics were presented in tabular form (Table 1), while quality appraisal results were illustrated using a visual summary (Figure 2). No sensitivity analyses were performed, as the limited number of included studies and their descriptive nature did not allow for comparative or stratified analyses.

A list of excluded studies after full-text review in Phase 2, along with reasons for exclusion, is provided in Appendix A.

### 2.8. Registration and Protocol

This systematic review was not registered in any publicly available database. No formal protocol was prepared prior to conducting the review. Consequently, no amendments to protocol or registration were made.

## 3. Results

The literature search yielded a total of 467 records after applying publication year filters. Following the removal of 55 duplicate entries, 412 articles were assessed during Phase 1 screening (title and abstract). Of these, 54 were selected for full-text review (Phase 2). Ultimately, 10 studies met all inclusion criteria and were incorporated into the systematic analysis.

### 3.1. Characteristics of the Included Studies

All included studies used autologous oral mucosa, either buccal, palatal, or labial, as a graft or flap for the reconstruction of defects in the nasal, septal, or sinonasal region.

Among the selected studies, five were individual case reports [2,5,8,18,19], and three were case series involving seven patients [1] and eight patients [20,21], respectively. Two studies were retrospective cohort analyses with 37 patients [6] and 61 patients [22]. Specifically, in the study by Kehrer et al. [22], only 29 patients were relevant to our review, as the remaining 32 formed a control group in which oral mucosa was not used for nasal mucosal reconstruction.

All included studies were case series or individual clinical case reports without a comparator group using alternative reconstructive techniques. Although they document favorable clinical outcomes with the use of oral mucosa grafts in nasal and sinonasal reconstruction, none of the studies provide direct evidence comparing their efficacy to other reconstructive methods, such as regional flaps, synthetic materials, or grafts from different donor sites. This methodological limitation prevents quantitative comparisons or definitive hierarchical conclusions regarding the superiority of oral mucosal grafts and highlights the need for prospective controlled studies to evaluate their performance relative to other therapeutic options. None of the included studies reported sources of funding or conflicts of interest. The characteristics of the included studies are summarized in Table 1.

**Table 1:** Characteristics of the included studies in the systematic review.

Author (Year)	Type of Study	No of Patients	Type of Patients (Population)	Graft	Intervention	Anatomical Area Treated	Clinical Outcomes	Complications
Kogan et al. (2007) [1]	Retrospective case series	7	Patients with septal perforation (post-septoplasty or tumor resection)	Pedicled	Oral mucosal flap via intentional oronasal fistula (sublabial, adjacent to upper labial frenulum)	Nasal septum	71.4% complete closure without rejection (n = 5)	Mild temporary difficulty with prosthetic denture adaptation (n = 1); complete flap loss (n = 2)
Kashiwa et al. (2009) [20]	Prospective case series	8	Patients with post-surgical or post-traumatic nasal mucosal defects	Pedicled	Sublabial myomucosal flap (orbicularis muscle + oral mucosa)	Nasal alar mucosa (n = 4), nasal floor (n = 2), columella (n = 2)	Viable flaps; good aesthetic and functional outcomes	Temporary upper lip hypoesthesia (n = 1); flap loss requiring secondary surgery (n = 2)
Biglioli et al. (2010) [18]	Case report	1	Patient with sinus membrane perforation (pre-dental implant)	Free	Free fibromucosal graft from hard palate	Maxillary sinus	Complete integration without complications	None at donor or recipient site
Gruber et al. (2015) [19]	Case report	1	Subtotal nasal resection due to squamous cell carcinoma	Pedicled	Pedicled rotational palatal flap	Nasal mucosa	Successful lining, no visible scar	None at donor or recipient site
Agrawal et al. (2016) [8]	Case report	1	Columellar loss, right nasal cavity stenosis, and aberrant scarring of alar cartilages post burn	Pedicled	Pedicled upper labial oral mucosal flap via oronasal fistula covered with skin graft	Nasal columella	Acceptable aesthetic result, no scarring	None at donor or recipient site
Feldman et al. (2017) [6]	Retrospective case series	37	Patients with septal perforation	Pedicled	Oral mucosal flap from upper labial sulcus pedicled via intentional oronasal fistula	Nasal septum	76% complete closure (n = 28)	24% (n = 9) developed oronasal fistula managed by oral edge approximation and suturing
Shin et al. (2018) [8]	Case report	1	78-year-old patient with sinus bone defect due to sinonasal mucosal melanoma	Free	Buccal mucosa + buccal fat	Maxillary sinus and nasal cavity	Complete defect closure	None at donor or recipient site
Kehrer et al. (2018) [22]	Retrospective case series	29	Cleft lip undergoing secondary rhinoplasty	Free	Buccal mucosa from inner cheek	Nasal mucosa	Aesthetic and functional nasal improvement	Transient mild infections or healing disturbances not requiring treatment
Kalmar et al. (2021) [21]	Prospective case series	8	Palatal fistulas and oronasal communication	Pedicled	Myomucosal flap from inner cheek	Nasal surface of palate	Successful closure (n = 7)	Incomplete closure (n = 1) required second surgery
Pitak-Arnnop et al. (2024) [5]	Case report	1	Complex naso-orbital trauma from chainsaw accident	Free	Buccal mucosa from inner cheek	Nasal mucosa	Complete integration with good functional and aesthetic result	None at donor or recipient site

According to the JBI assessment, the five individual case reports [2,5,8,18,19] generally demonstrated moderate methodological quality. One of them presented a high risk of bias [19], three presented a moderate risk [2,8,18], and one presented a low risk [5]. However, three studies [8,18,19] presented limitations in the description of follow-up, reducing the ability to assess the long-term stability of the results.

As for the five case series [1,6,20,21,22], the studies by Kehrer et al. [22], Kashiwa et al. [20], and Kalmar et al. [21] were considered to have moderate risk of bias. One of them was classified as low risk [1] and another one as moderate-high risk [6] according to the Joanna Briggs Institute criteria. The studies by Feldman et al. [6] and Kehrer et al. [22] showed certain weaknesses, such as insufficient documentation of follow-up [6], incomplete description of inclusion criteria [6], or other methodological issues [22].

Overall, all studies contribute clinically useful evidence, although it is inherently limited by the study designs. The findings were consistent across most studies, despite methodological heterogeneity and moderate overall quality. A full summary of the synthesized outcomes and risk of bias is presented in Table 1 and Figure 2.

### 3.2. Treated Anatomical Sites

The anatomical sites reconstructed included the nasal septum [1,6], nasal columella [8], maxillary sinus and nasal cavity [2,18], nasal mucosa [5,19,20,22], and the nasal surface of the hard palate [21]. This highlights the versatility and anatomical adaptability of oral mucosal grafts across various locations within the sinonasal region.

### 3.3. Techniques and Types of Grafts/Flaps

Both pedicled [1,6,8,19,20,21] and free [2,5,18,22] oral mucosal grafts were used. Various surgical techniques were employed, including the superior sublabial oral mucosal flap [1,6,8,20], rotational palatal flap [19], myomucosal flap from the inner cheek [21], free palatal graft [18], free buccal mucosa with buccal fat [2], and free buccal mucosa without fat [5,22].

In all cases, autologous tissue was used, with no reliance on synthetic materials.

### 3.4. Clinical Outcomes and Complications

All studies reported high clinical success rates, with complete defect closure and satisfactory functional and/or aesthetic outcomes.

Only four studies reported complications. Kogan et al. [1] described a single case of mild transient difficulty (2–3 months) with prosthetic denture adaptation. Kashiwa et al. [20] reported temporary upper lip hypoesthesia in 12.5% of patients (n = 1). In the study by Feldman et al. [6], 24% of patients (n = 9) developed oronasal fistulas, though none experienced graft loss or major complications.

The reported rates of complete flap loss or incomplete nasal defect closure in multi-patient series were as follows: 28.57% (n = 2) in Kogan et al. [1], 25% (n = 2) in Kashiwa et al. [20], 24.32% (n = 9) in Feldman et al. [6], and 12.5% (n = 1) in Kalmar et al. [21].

No graft rejection, serious infections, or flap necrosis were observed in the remaining studies during follow-up.

Among the 10 included studies, only Kogan et al. [1] and Kashiwa et al. [20] reported donor site morbidity, specifically a transient difficulty with denture adaptation and upper lip hypoesthesia, respectively, both of which resolved over time.

### 3.5. Clinical Follow-Up

Considerable variability was observed in the follow-up periods across the included studies. Two studies reported structured follow-up: Kogan et al. [1] with a mean of 24 months and Pitak-Arnnop et al. [5] with a 3-month follow-up. In the remaining case reports, follow-up was either short or not clearly specified. Feldman et al. [6] mentioned mid-term follow-up, although the exact duration was not reported. Kalmar et al. [21] described short-to-mid-term follow-up (mean of 5 months), while Kashiwa et al. [20] and Kehrer et al. [22] reported longer follow-up periods, ranging from 10 to 70 months and up to 97 months, respectively.

Overall, clinical follow-up data are limited; however, in cases where follow-up was documented, the graft demonstrated good long-term functional stability.

### 3.6. Synthesis of Results

The included studies were predominantly case reports and small case series, reflecting limited and heterogeneous evidence on the use of oral mucosal grafts in rhinological and skull base reconstruction. All studies involved the use of either pedicled or free oral mucosa grafts for reconstructing defects in varied anatomical regions, including the nasal septum, columella, nasal floor, and anterior skull base.

Due to the heterogeneity in study design, patient populations, graft techniques, and reported outcomes, a meta-analysis was not feasible. Instead, a narrative synthesis was performed. The overall success rate was high across studies, with most cases reporting satisfactory graft integration and minimal complications. Complications, when reported, were minor and included temporary donor site hypoesthesia or mild flap loss.

Considerable clinical heterogeneity was noted regarding graft type (free vs. pedicled), anatomical area treated, and underlying pathology. These differences limit the comparability of outcomes and make it difficult to generalize conclusions.

### 3.7. Relevant Observations

Buccal mucosa was the most used graft type, as reported in 8 of the 10 studies analyzed, and was applied both as a free graft and as a flap. In contrast, Gruber et al. [19] and Biglioli et al. [18] opted for palatal mucosa, emphasizing its advantages in terms of thickness and structural resistance.

Regarding the recipient anatomical site, oral mucosal grafts proved effective both in structurally complex areas, such as the columella, and in internal mucosal regions, such as the nasal septum and maxillary sinus.

Finally, all included studies reported good postoperative tolerance, with no severe or permanent sequelae at the donor site (oral cavity).

Due to the descriptive and heterogeneous nature of the included case reports and series, most studies did not report numerical outcome measures or comparative statistics. Where available, information on graft viability, complications, and follow-up duration was extracted and summarized narratively.

## 4. Discussion

Although the studies included in this systematic review provide limited scientific evidence, they collectively support a positive stance toward the use of oral mucosal grafts as a reconstructive alternative for the nasal septum, nasal cavities, paranasal sinuses, and the anterior skull base. Across these indications, outcomes were favorable in terms of function, aesthetics, and scarring, with few complications reported, mainly transient oedema or mild discomfort at the donor site. No significant graft rejection or necrosis was observed. Although follow-up duration varied among studies, most reported graft survival and functional epithelialization in the short to mid-term. Taken together, these findings support the view that oral mucosa is a versatile and functional graft material [1,4,11,23].

According to the published studies, oral mucosal grafts offer numerous advantages, providing a viable alternative in settings where a stable lining with good adaptability to the nasal or sinus environment is required [10]. As an autologous tissue, it is easy to harvest, well vascularized, and capable of integrating effectively into moist cavities, particularly the nasal cavity [24,25].

In other anatomical fields such as urology, oral mucosa is widely used with high success rates in urethroplasties [11,24,26] and ureteroplasties [26]. In gynecologic surgery, it is employed in vaginoplasty procedures for patients with Mayer–Rokitansky–Küster–Hauser syndrome or in gender-affirming surgeries, where oral mucosa offers a lubricated hairless mucosal tissue with thickness and elasticity similar to vaginal mucosa [27]. For this reason, oral mucosa may be considered a valid alternative in genital reconstruction when other options are unavailable or suboptimal [27].

In ophthalmology, oral mucosa has been used for conjunctival and fornix reconstruction, particularly in severe cicatricial diseases. Although it lacks goblet cells, its ease of harvest in sufficient volume makes it highly advantageous when autologous conjunctiva is insufficient [23]. Once integrated, it provides a stable non-keratinized surface, improving ocular motility and reducing scarring recurrence [23].

In otolaryngology and head and neck surgery, the use of oral mucosa as a free graft has primarily been documented in scenarios where nasal mucosa is absent or damaged. One of the most representative indications is the reconstruction of septal perforations, with reported reperforation rates ranging from 12.5% to 28.6% [1,6,20,22]. In cases involving large perforations or diseased nasal mucosa, a pedicled buccal mucosal flap was used via the gingivolabial sulcus, yielding favorable functional outcomes and low morbidity [1]. Shin et al. [2] reported positive results using pedicled oral mucosal flaps combined with buccal fat for reconstruction of the nasal cavity and maxillary sinus.

Similarly, Wu et al. [3] described the combined use of a cutaneous forehead flap and palatal mucosa for nasal and facial reconstruction in patients with extensive carcinomas. In nasolacrimal surgery, grafting oral mucosa around the ostium edges during dacryocystorhinostomy helped maintain ostium patency in patients with refractory acquired obstruction, particularly those with atrophic rhinitis or insufficient healthy nasal mucosa [3].

In oncologic contexts, oral mucosa has also been used as a free graft to line nasal cavities following extensive mucosal resections [3] with favorable outcomes. Likewise, in the repair of anterior skull base cerebrospinal fluid leaks, oral mucosa may serve as a valid alternative when autologous nasal mucosa is unavailable [16].

Additionally, oral mucosa has been applied in extranasal locations, such as in tracheal and laryngeal reconstruction for severe subglottic stenosis, where it was used to line the expanded tracheal lumen [17]. In these cases, oral mucosa helped prevent granulation tissue formation and reduce restenosis, allowing for long-term airway patency [17].

Several studies have proposed the use of biomaterials as an alternative to autologous tissue. The use of acellular dermal matrix [28] or collagen–elastin matrix [28] has shown promising results in the closure of complex oronasal fistulas, avoiding the need for grafts and reducing donor site morbidity. However, their application in nasal, septal, or sinonasal reconstruction remains limited in the literature and differs significantly from the biological behavior of oral mucosa.

Other authors have used amniotic membrane [28] or resorbable magnesium membrane [29] for closing septal or sinus perforations. Although these strategies eliminate the need for autologous tissue, they require specific synthetic materials, which may limit their applicability in certain clinical settings for various reasons.

Moreover, the development of new pedicled nasal flaps, combined with advancements in endoscopic sinonasal surgery, has gradually shifted interest away from biomaterials in favor of local flaps for the closure of septal perforations [30,31] or for oroantral fistulas [32].

It is worth emphasizing that, despite the availability of various reconstructive alternatives, oral mucosa may represent a viable option in cases where nasal mucosa is absent or insufficient. Its autologous nature, immediate availability, and ease of handling make it a versatile alternative in settings where local tissue is of poor quality or inadequate in volume. Furthermore, its biological compatibility is particularly high in the sinonasal and skull base regions [3,13,28,29,33,34,35,36]. Recent studies on regenerative surgery and mucoplasty, particularly in the context of surgical treatment for chronic rhinosinusitis with nasal polyps, have renewed interest in identifying donor sites with healthy non-pathological mucosa beyond the nasal mucosa itself [7].

The studies analyzed reported no major complications, with the most frequent being transient hypoesthesia at the donor site [1,6,8]. No significant rates of graft rejection, serious infection, or flap necrosis were described during follow-up.

It is important to note that the methodological quality of the ten included studies presents significant limitations. All analyzed publications were case series or individual clinical case reports, lacking comparison groups with other reconstructive techniques and generally retrospective in nature, which limits the strength of the evidence. Kehrer et al. [22] compared the use of oral mucosa in sinonasal reconstruction with a control group that did not undergo reconstruction, a noteworthy approach given current trends advocating this strategy in certain pathologies such as chronic rhinosinusitis with nasal polyps.

The certainty of the evidence was not formally assessed using tools such as GRADE, given the descriptive design and non-comparative nature of the included studies. This represents a limitation of the review and highlights the need for higher-quality comparative research in this area.

Limitations of the review process include the absence of a protocol registration and the fact that study selection and data extraction were primarily conducted by a single reviewer, which may introduce selection bias. Additionally, the heterogeneity of the included studies limited the ability to perform formal synthesis or subgroup analysis.

The potential for publication bias could not be formally assessed, as no quantitative synthesis was performed and all included studies were descriptive. However, the predominance of case reports and small case series with favorable outcomes suggests a high risk of reporting bias.

In addition, considerable heterogeneity was observed in the surgical techniques used, the anatomical sites treated, and the clinical follow-up reported, making cross-study comparisons difficult. Although some series [1,6,20,21,22] provide more extensive data, the overall level of evidence remains low. Nonetheless, the systematic application of JBI tools confirmed that the included studies clearly described the interventions and clinical outcomes. The most common methodological weakness was limited long-term follow-up; with the exception of two studies [20,22], follow-up was generally under 12 months.

Another important limitation is that none of the included studies objectively assessed patients’ quality of life following reconstruction with oral mucosa using validated questionnaires. Although some authors reported subjective clinical improvements, such as relief from nasal obstruction or resolution of crusting, these observations do not allow for a meaningful evaluation of the treatment’s functional or psychosocial impact. The absence of quality-of-life assessment represents a significant gap in the current literature and should be considered a priority for future clinical research. Therefore, prospective comparative studies and controlled clinical trials are needed to evaluate the safety and efficacy of these grafts.

The findings of this review, therefore, support the potential use of oral mucosa as a reconstructive option for septal, sinonasal, or skull base defects. Future research should also investigate differences between types of oral mucosa (buccal, fatty, palatal) and adopt longer follow-up periods to assess long-term outcomes and graft viability across various anatomical sites.

## 5. Conclusions

Oral mucosal grafts and flaps represent an effective and viable reconstructive option for the repair of nasal, septal, sinonasal, and skull base defects, particularly in cases where local mucosa is insufficient, absent, or pathologic.

The observed advantages include the immediate availability of the graft, the potential for harvesting sizable tissue, ease of handling, good donor site tolerance, and excellent adaptability to recipient sites. Reported complications are mostly local and temporary.

Prospective studies with larger sample sizes, control groups, and comparative designs are needed to rigorously assess the efficacy and safety of oral mucosal grafts in these anatomical regions.

## Data Availability

No datasets or analytic code were generated. Data extraction tables and screening forms used in this systematic review are available from the corresponding author upon reasonable request.

## Abbreviations

The following abbreviations are used in this manuscript:

JBI	Joanna Briggs Institute
FESS	Functional Endoscopic Sinus Surgery

## Appendix A. Excluded Studies After Full-Text Review

**Study (First Author, Year)**	**Reason for Exclusion**
Fan et al. (2005)	Language other than English or Spanish
Liu et al. (2006)	Grafts unrelated to oral mucosa/non-reconstructive intent
Zhan et al. (2006)	Language other than English or Spanish
Katranji et al. (2008)	Grafts unrelated to oral mucosa/non-reconstructive intent
Hernandez-Alfaro et al. (2008)	No description of clinical or surgical outcomes
Kirmeier et al. (2008)	No description of clinical or surgical outcomes
Meirelles et al. (2008)	Unrelated recipient area
Kim et al. (2008)	Document type not eligible
El Marakby et al. (2008)	Full text unavailable
Hassani et al. (2009)	Grafts unrelated to oral mucosa/non-reconstructive intent
Zhang et al. (2010)	Grafts unrelated to oral mucosa/non-reconstructive intent
Kinnunen et al. (2010)	Grafts unrelated to oral mucosa/non-reconstructive intent
Yalçin et al. (2011)	Unrelated recipient area
Moon et al. (2011)	Grafts unrelated to oral mucosa/non-reconstructive intent
Patrocinio et al. (2011)	Grafts unrelated to oral mucosa/non-reconstructive intent
Mai et al. (2013)	Unrelated recipient area
Benateau et al. (2014)	Language other than English or Spanish
Durr et al. (2014)	Grafts unrelated to oral mucosa/non-reconstructive intent
Coronel-Banda et al. (2014)	Grafts unrelated to oral mucosa/non-reconstructive intent
Gun et al. (2014)	Document type not eligible
Colletti et al. (2014)	Grafts unrelated to oral mucosa/non-reconstructive intent
Fang et al. (2014)	Unrelated recipient area
El-Kassaby et al. (2014)	Grafts unrelated to oral mucosa/non-reconstructive intent
Adams et al. (2015)	Unrelated recipient area
Polyakov et al. (2015)	Document type not eligible
Marcantonio et al. (2015)	Unrelated recipient area
Noel et al. (2016)	Grafts unrelated to oral mucosa/non-reconstructive intent
Sohail et al. (2016)	Unrelated recipient area
Prince et al. (2016)	Grafts unrelated to oral mucosa/non-reconstructive intent
Salcedo-Gil et al. (2017)	Document type not eligible
Sharma et al. (2017)	Grafts unrelated to oral mucosa/non-reconstructive intent
Zenga et al. (2017)	No description of clinical or surgical outcomes
Darr et al. (2018)	Grafts unrelated to oral mucosa/non-reconstructive intent
Stavropoulou et al. (2018)	Unrelated recipient area
Wu et al. (2018)	Document type not eligible
Basagaoglu et al. (2018)	No description of clinical or surgical outcomes
Mannino et al. (2020)	Unrelated recipient area
Lee et al. (2021)	Document type not eligible
Amnion et al. (2022)	Grafts unrelated to oral mucosa/non-reconstructive intent
Vale et al. (2022)	Document type not eligible
Elad et al. (2023)	Grafts unrelated to oral mucosa/non-reconstructive intent
Nisenbaum et al. (2023)	Document type not eligible
Do et al. (2024)	Grafts unrelated to oral mucosa/non-reconstructive intent
Yu et al. (2025)	No description of clinical or surgical outcomes
